# A paralogous pair of mammalian host restriction factors form a critical host barrier against poxvirus infection

**DOI:** 10.1371/journal.ppat.1006884

**Published:** 2018-02-15

**Authors:** Xiangzhi Meng, Fushun Zhang, Bo Yan, Chuanping Si, Hiroaki Honda, Akiko Nagamachi, Lu-Zhe Sun, Yan Xiang

**Affiliations:** 1 Department of Microbiology, Immunology and Molecular Genetics, University of Texas Health Science Center at San Antonio, San Antonio, Texas, United States of America; 2 Institute of Immunology and Molecular Medicine, Jining Medical College, Jining, Shandong, China; 3 Institute of Laboratory Animals, Tokyo Women’s Medical University, Shinjuku-ku, Tokyo, Japan; 4 Department of Molecular Oncology and Leukemia Program Project, Hiroshima University, Minami-ku, Hiroshima, Japan; 5 Department of Cell Systems & Anatomy, University of Texas Health Science Center at San Antonio, San Antonio, Texas, United States of America; Ludwig-Maximilians-Universität München, GERMANY

## Abstract

Host restriction factors constitute a formidable barrier for viral replication to which many viruses have evolved counter-measures. Human SAMD9, a tumor suppressor and a restriction factor for poxviruses in cell lines, is antagonized by two classes of poxvirus proteins, represented by vaccinia virus (VACV) K1 and C7. A paralog of SAMD9, SAMD9L, is also encoded by some mammals, while only one of two paralogs is retained by others. Here, we show that SAMD9L functions similarly to SAMD9 as a restriction factor and that the two paralogs form a critical host barrier that poxviruses must overcome to establish infection. In mice, which naturally lack SAMD9, overcoming SAMD9L restriction with viral inhibitors is essential for poxvirus replication and pathogenesis. While a VACV deleted of both K1 and C7 (vK1L^-^C7L^-^) was restricted by mouse cells and highly attenuated in mice, its replication and virulence were completely restored in *SAMD9L*^*-/-*^ mice. In humans, both SAMD9 and SAMD9L are poxvirus restriction factors, although the latter requires interferon induction in many cell types. While knockout of SAMD9 with Crispr-Cas9 was sufficient for abolishing the restriction for vK1L^-^C7L^-^ in many human cells, knockout of both paralogs was required for abolishing the restriction in interferon-treated cells. Both paralogs are antagonized by VACV K1, C7 and C7 homologs from diverse mammalian poxviruses, but mouse SAMD9L is resistant to the C7 homolog encoded by a group of poxviruses with a narrow host range in ruminants, indicating that host species-specific difference in SAMD9/SAMD9L genes serves as a barrier for cross-species poxvirus transmission.

## Author summary

Zoonotic viral infections represent a major threat to public health. For many viruses, host species-specific difference in viral entry receptors presents a major hurdle for cross-species transmission. Poxviruses, however, can enter nearly any animal cell. Why many poxviruses show strict host species specificity and what it would take for them to jump to new hosts are less clear. Here, we present data suggesting that SAMD9 and its paralog, SAMD9L, constitute a critical host barrier against poxvirus infection and pathogenesis. We also discovered some host species-specific difference in SAMD9/SAMD9L and some poxvirus-specific difference in antagonizing SAMD9/SAMD9L, suggesting that these differences serve as a barrier for cross-species poxvirus infection. The knowledge is fundamental for understanding the determinants of poxvirus host-range.

## Introduction

Emerging and reemerging infectious diseases have continued to pose a major threat to public health. In particular, zoonotic viral infections have caused such lethal human diseases as SARS, avian influenza, human monkeypox, and Ebola [1]. For many viruses, including coronaviruses and influenza viruses, host species-specific difference in viral entry receptors presents a major hurdle for cross-species transmission [2]. Poxviruses, however, can enter nearly any animal cell [3]. Why many poxviruses show strict host species specificity and what it would take for them to jump to new hosts are less clear [4]. Poxviruses include many lethal animal and human pathogens [5], the most infamous of which is the smallpox-causing variola virus. Smallpox was successfully eradicated mainly through a global immunization program with vaccinia virus (VACV), and routine VACV vaccination had since discontinued. The human population is now vulnerable to zoonotic orthopoxvirus infection, as some extant poxviruses related to variola virus are capable of infecting a wide variety of wild and domestic animals. There are also many poxviruses with a more restricted host range [6]. For example, capripoxviruses, consisting of sheeppox virus, goatpox virus, and lumpy skin disease virus, have a very narrow host-range in ruminants, causing economically significant diseases in sheep, goats, and cattle, respectively. Host-restricted poxviruses have been exploited as safe vectors for vaccines, gene therapy or oncolytic viral therapies, although the basis for their host restriction is largely unknown [4].

Poxvirus host range at the cellular level is governed by a group of poxvirus genes referred to as the host range genes [4, 6]. The first discovered and perhaps the most important host range genes are K1L and C7L of VACV [7, 8]. VACV replication in most mammalian cell lines requires either K1L or C7L [7], and the deletion of both genes from VACV aborts the replication prior to viral late gene expression [9]. K1L is only present in VACV and a few related orthopoxviruses, but a C7L homolog that functions nearly identically to VACV C7L is present in most mammalian poxviruses [10]. SAMD9 (Sterile Alpha Motif Domain-containing 9) was found to be the restriction factor in human cell lines that blocked the replication of poxvirus mutants that lack K1L and C7L-like genes [11]. K1 and C7 are structurally distinct [12, 13], but K1 and many C7 homologs independently bind and inhibit human SAMD9 [11, 12, 14].

SAMD9 was initially identified as a tumor suppressor whose loss-of-function mutations in humans cause normophosphatemic familial tumoral calcinosis (NFTC) [15, 16]. Humans and many other mammals also encode a chromosomally adjacent paralog of SAMD9 named SAMD9-like (SAMD9L). Human SAMD9 and SAMD9L are ubiquitously expressed in many tissues [17], and their expression can be further induced by interferons [18, 19]. Evolutionary analysis suggested that the two paralogs derived from the duplication of an ancestral gene early in mammalian evolution and that some mammalian species suffered a loss of either SAMD9 or SAMD9L [20]. Notably, mice lack SAMD9, while many ruminants lack SAMD9L. Mouse SAMD9L is also a tumor suppressor, and haploinsufficiency of mouse SAMD9L resulted in myeloid malignancies [21]. The molecular functions of SAMD9 family of proteins remain largely elusive, but recent sequence analysis predicted a complex domain architecture suggestive of a regulative function of a putative signal transduction network [22].

Although SAMD9 has been established as a critical poxvirus restriction factor in human cell lines, whether this restriction is important at the organismal level and in other mammalian species is unknown. Moreover, whether SAMD9L plays a role in host defense is unknown. We studied the function of SAMD9L from a host that lacks SAMD9 (mice) as well as a host that maintains both paralogs (humans). We found that SAMD9L functions nearly identically to SAMD9 as a host restriction factor and that overcoming SAMD9/SAMD9L (SAMD9/L) restriction is essential for poxvirus replication and pathogenesis. We also discovered some host species-specific difference in SAMD9/L and some poxvirus species-specific difference in antagonizing SAMD9/L, suggesting that these differences contribute to the barrier for cross-species poxvirus infection.

## Results

### Mouse SAMD9L is the restriction factor for K1L and C7L deletion vaccinia virus in mouse cells

**Fig 1:**
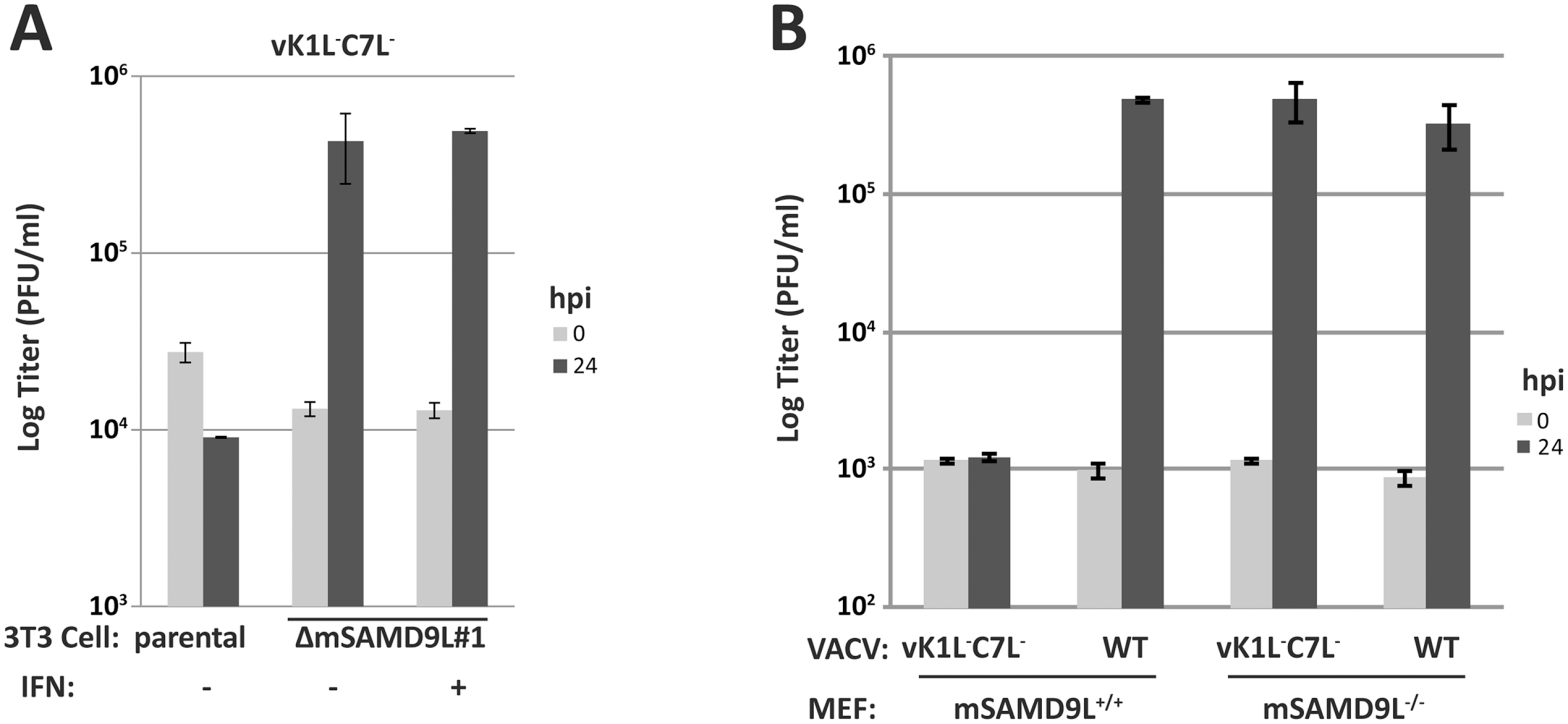
mSAMD9L is a poxvirus restriction factor that needs to be overcome for poxvirus replication. **(A)**. The restriction of K1L and C7L deletion vaccinia virus (vK1L^-^C7L^-^) in 3T3 cells was abolished by knocking out mSAMD9L with CRISPR-Cas9. A validated mSAMD9L knockout cell clone (ΔmSAMD9L #1) and the parental 3T3 cells were left untreated (-) or treated (+) with 200 U/ml of murine IFN-β for 24 h, before they were infected with vK1L^-^C7L^-^ at an MOI of 1 PFU/cell. Viral growth was determined by measuring viral titers at 0 and 24 hour-post-infection (hpi). **(B)**. The restriction of vK1L^-^C7L^-^ in mouse embryo fibroblasts (MEF) was abolished by knocking out mSAMD9L. MEFs isolated from *SAMD9L*^*+/+*^ or *SAMD9L*^*-/-*^ mice were infected with vK1L^-^C7L^-^ or wild-type (WT) VACV strain WR.

The importance of human SAMD9 as a poxvirus restriction factor became evident only when viral inhibitors of SAMD9 were removed from poxviruses, so we used a VACV mutant deleted of both K1L and C7L (vK1L^-^C7L^-^) as the model poxvirus to study poxvirus restriction factors in different host species. Mouse is one of the mammalian species that have lost *SAMD9* gene, and a previous study suggested that mouse SAMD9L was not a functional paralog of human SAMD9 in terms of the tumor suppressor function [23]. However, mouse cells, such as NIH 3T3 cells and mouse embryonic fibroblasts (MEFs), restricted the replication of vK1L^-^C7L^-^ [24]. To test whether mouse SAMD9L (mSAMD9L) might be the restriction factor for vK1L^-^C7L^-^, we used the CRISPR-Cas9 technology to knock out mSAMD9L from 3T3 cells. To control for potential off-target effect, we performed two independent knockouts with different guide sequences. To confirm the gene knockout, cell clones were isolated, and the mSAMD9L genotype of representative clones from the two knockouts (named ΔmSAMD9L#1 and ΔmSAMD9L#2) was determined by sequencing. All mSAMD9L alleles of the cell clones were found to contain indels, resulting predominantly in frameshift (S1A Fig). While vK1L^-^C7L^-^ was unable to replicate in the parental 3T3 cells, it replicated well in ΔmSAMD9L#1 (Fig 1A) and ΔmSAMD9L#2 (S1B Fig) cells, resulting in nearly 100-fold increase of viral titer after 24 hours of infection. Moreover, while vK1L^-^C7L^-^ was sensitive to interferons (IFNs) in permissive human cells [24, 25], its growth in ΔmSAMD9L cells was not inhibited by pretreating the cells with IFN-β (Fig 1A).

As an alternative to the CRISPR-Cas9 knockout, we prepared MEFs from *SAMD9L*^*+/+*^ and *SAMD9L*^*-/-*^ mice. While vK1L^-^C7L^-^ failed to grow in *SAMD9L*^*+/+*^ MEFs, its replication was completely restored in *SAMD9L*^*-/-*^ MEFs (Fig 1B), confirming that mSAMD9L is the restriction factor for vK1L^-^C7L^-^ in mouse cells.

### Mouse SAMD9L constitutes an essential host barrier for vK1L^-^C7L^-^ replication and pathogenesis

**Fig 2:**
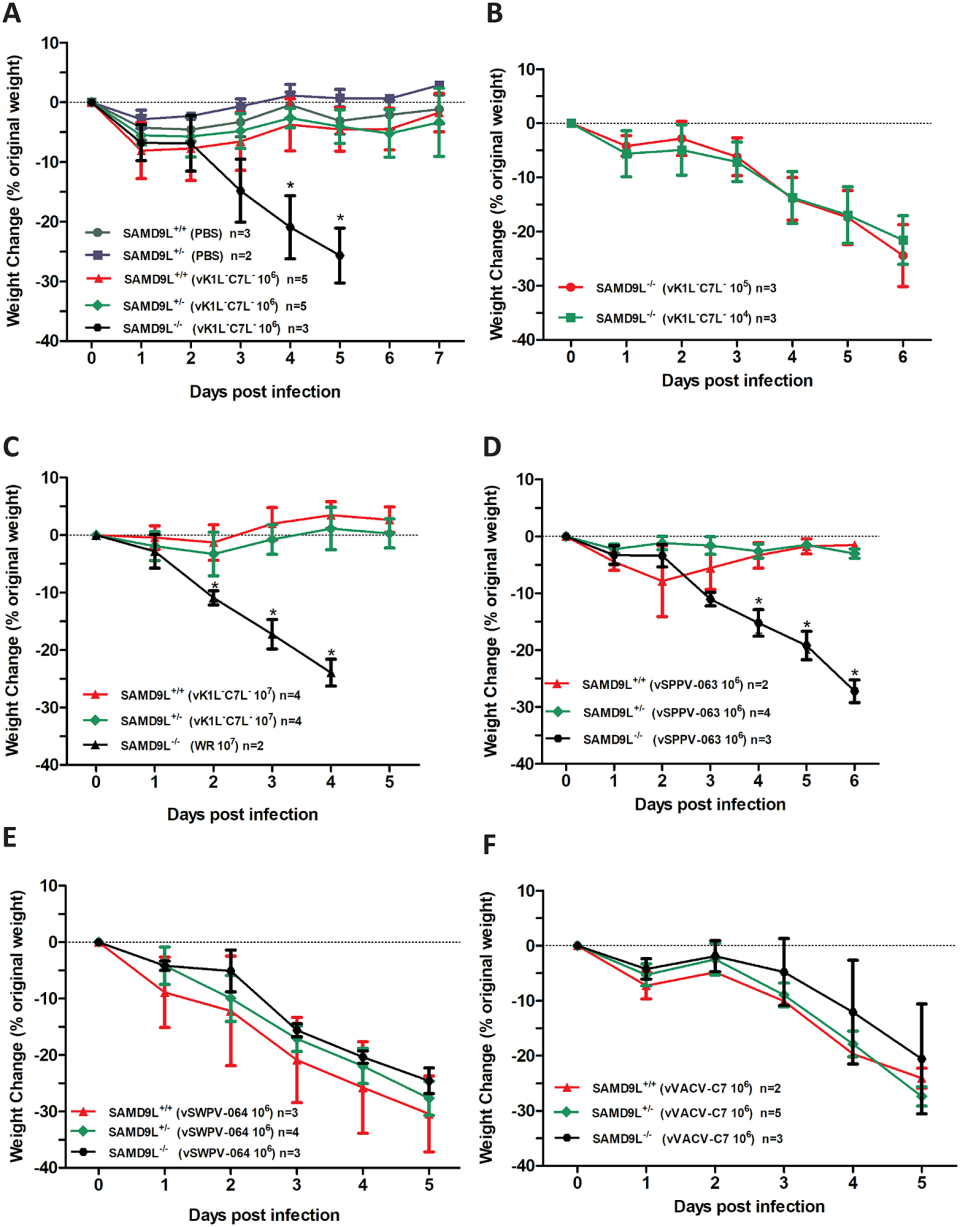
mSAMD9L is a poxvirus restriction factor that needs to be overcome for poxvirus pathogenesis. **(A, B, C)**. vK1L^-^C7L^-^ virulence was restored in *SAMD9L*^*-/-*^ mice. Littermates from *SAMD9L*^*+/-*^ mice were grouped according to their SAMD9L genotypes and infected intranasally with vK1L^-^C7L^-^ or mock infected (PBS). The body weight change following the infection (average and standard deviation) is shown for each group. “*” indicates statistically significant difference (p <0.05 by two way ANOVA analysis). n denotes group size. WT VACV WR strain was used as a control in *C*. **(D,E,F)**. The C7 homolog from sheeppox virus (SPPV-063) has a host-species specific defect in mice. Same as *A* except the viruses used were vSPPV-063 **(D)**, vSWPV-064 **(E)**, or vVACV-C7 **(F)**, which are vK1L^-^C7L^-^-derived viruses that expressed SPPV-063, SWPV-064 or VACV-C7, respectively.

vK1L^-^C7L^-^ is highly attenuated in mice [10]. To determine whether mSAMD9L functions as a restriction factor at the organismal level, we tested whether vK1L^-^C7L^-^ would regain virulence in *SAMD9L*^*-/-*^ mice. Intranasal infection of *SAMD9L*^*+/+*^ and *SAMD9L*^*+/-*^ mice with 10^6^ plaque-forming unit (PFU) of vK1L^-^C7L^-^ did not cause any disease symptom or sustained body weight loss, similar to the mock infection (Fig 2A). The infected mice developed an antibody response to VACV (S2A Fig), indicating that they were properly infected. In contrast to their *SAMD9L*^*+/+*^ and *SAMD9L*^*+/-*^ littermates, all *SAMD9L*^*-/-*^ mice lost close to 25% of body weight by day 5 post infection and had to be euthanized. A similar lethal effect on *SAMD9L*^*-/-*^ mice was observed when the infectious dosage was reduced to 10^5^ or 10^4^ PFU (Fig 2B), indicating that vK1L^-^C7L^-^ was highly virulent in *SAMD9L*^*-/-*^ mice.

In mice, SAMD9L haploinsufficiency caused myeloid malignancies [21]. To test whether SAMD9L haploinsufficiency would increase susceptibility to vK1L^-^C7L^-^ infection, we infected *SAMD9L*^*+/+*^ and *SAMD9L*^*+/-*^ mice with the highest dosage of vK1L^-^C7L^-^ we could reasonably obtain (10^7^ PFU in 20 μl). Again, neither group of mice developed any disease symptoms or had sustained body weight loss (Fig 2C), and no virus was detected in lungs when the mice were euthanized at five days post infection (S2B Fig), indicating that one copy of *mSAMD9L* gene is sufficient for restricting vK1L^-^C7L^-^.

Altogether, the results from SAMD9L knockout cells and mice demonstrate that mSAMD9L constitutes an essential host barrier for vK1L^-^C7L^-^ replication and pathogenesis.

### The C7 homolog from sheeppox virus has a host-species specific defect in mice

We previously used vK1L^-^C7L^-^ as the parental virus in constructing a panel of recombinant viruses that expressed different C7 homologs from representative mammalian poxviruses [10, 24]. This panel of viruses were used for comparing the function of different C7 homologs in the same viral background, and they served as a surrogate model for different mammalian poxviruses in terms of their ability to overcome SAMD9 restriction. Studies of these viruses showed that the C7 homologs from many mammalian poxviruses, including myxoma virus (MYXV; infect rabbits) MYXV-M62, Yaba-like diseases virus (YLDV; infect monkeys) YLDV-67, swinepox virus (SWPV) SWPV-064, and sheeppox virus (SPPV) SPPV-063, could bind human SAMD9 and overcome its restriction [12]. All these C7 homologs, except for SPPV-063, could also overcome the restriction of vK1L^-^C7L^-^ by mouse cell lines [24]. To determine whether the defect of SPPV-063 in mouse cell lines reflects a host species-specific defect, we studied the mouse virulence of vSPPV-063, the vK1L^-^C7L^-^-derived recombinant virus that expressed SPPV-063. Swinepox virus SWPV-064 is the closest homolog of SPPV-063, so vSWPV-064, the vK1L^-^C7L^-^-derived recombinant virus that expressed SWPV-064, was used as the control. Similar to vK1L^-^C7L^-^, vSPPV-063 did not result in significant body weight loss in *SAMD9L*^*+/-*^ or *SAMD9L*^*+/+*^ mice but caused lethal intranasal infection in *SAMD9L*^*-/-*^ mice (Fig 2D). In contrast, the same dose of vSWPV-064 was lethal to *SAMD9L*^*+/+*^ and *SAMD9L*^*+/-*^ mice as well as to *SAMD9L*^*-/-*^ mice (Fig 2E). In a separate experiment, vVACV-C7, the vK1L^-^C7L^-^-derived recombinant virus that expressed VACV-C7, was also lethal to *SAMD9L*^*+/+*^ and *SAMD9L*^*+/-*^ mice (Fig 2F). As SPPV-063 can overcome SAMD9 restriction in human cells, these data show that SPPV-063 has a host species-specific defect in mice.

### VACV K1, C7 and C7 homologs from diverse poxviruses could bind mSAMD9L, but the sheeppox virus C7 homolog has a specific defect due to the variation of two amino acids

**Fig 3:**
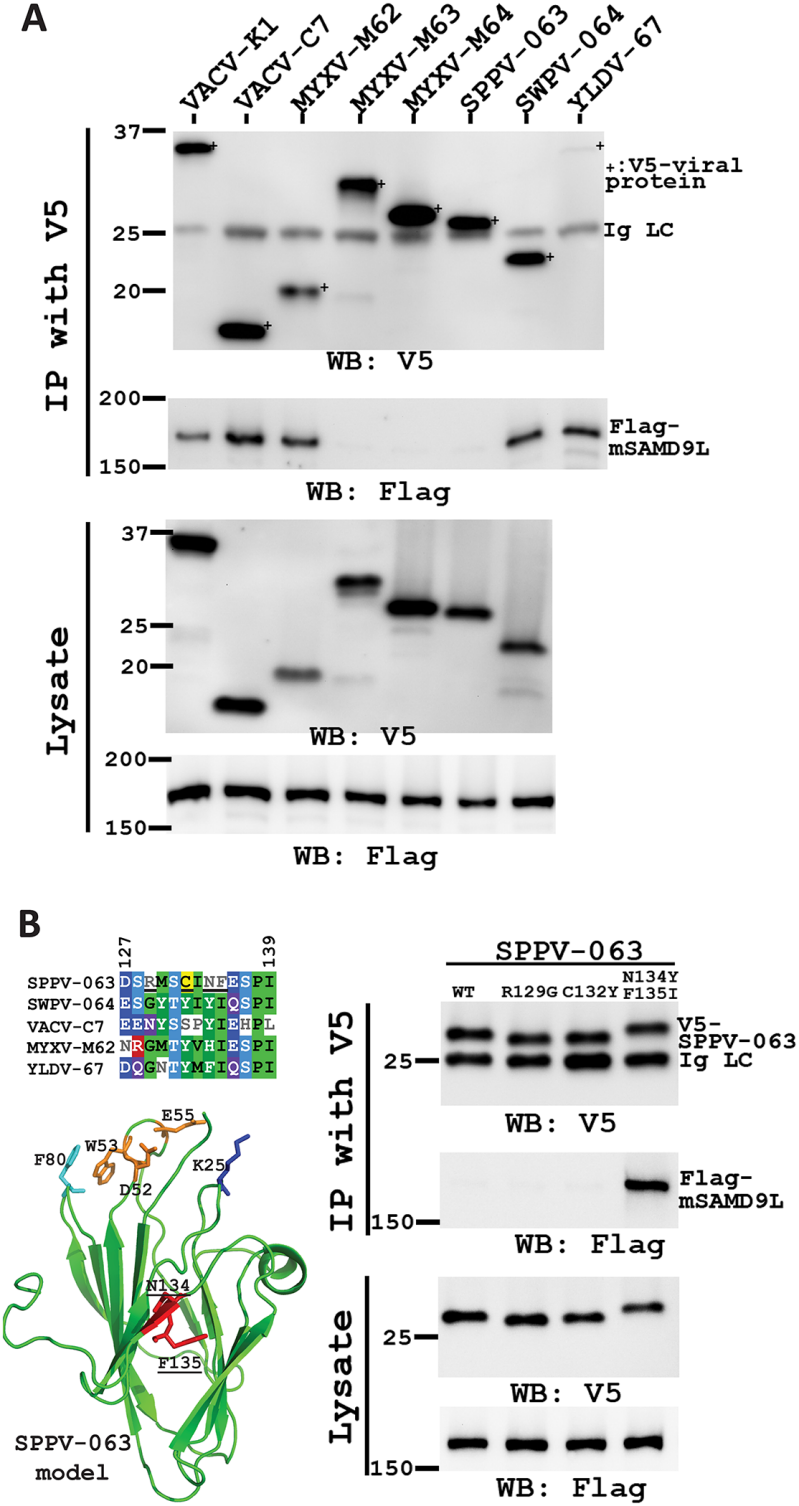
VACV K1, C7 and C7 homologs from diverse poxviruses could bind mSAMD9L, but the C7 homolog from sheeppox virus has a specific defect due to the variation of two amino acids. **(A)**. The sheeppox virus C7 homolog (SPPV-063) was defective of binding mSAMD9L. 293FT cells were transfected with a plasmid expressing flag-tagged mSAMD9L and infected with the panel of vK1L^-^C7L^-^-derived VACVs that expressed V5-tagged VACV-K1 or a C7 homolog from different poxviruses. The cell lysates were subjected to immunoprecipitation with an anti-V5 antibody. mSAMD9L and the epitope-tagged viral protein in the cell lysate and precipitate were detected respectively with anti-flag and anti-V5 antibody in Western blot. The light chain of the precipitated antibody (Ig LC) serves as a loading control. Due to space limitation, lysate of YLDV-67 sample was not loaded on the same gel. **(B)**. Substitution of residue 134 and 135 of SPPV-063 restored binding to mSAMD9L. WT SPPV-063 and mutants with substitution at residue 129 (R129G), 132 (C132Y), or both 134 and 135 (N134Y, F135I) were precipitated as described in *A*. A multiple sequence alignment of different C7 homologues encompassing the substituted region is shown. Residues with similar physicochemical properties among at least 4 of the homologs are highlighted and colored by their properties (hydrophobic, green; negative, bright blue; positive, red; polar, purple; cysteine, yellow; alcohol, light blue). A model of SPPV-063 based on the VACV-C7 structure is also shown with critical SAMD9-binding residues labeled.

A biochemical basis for SAMD9 antagonism by the poxvirus proteins is their binding of SAMD9 [12], so we next studied the binding of mSAMD9L by the viral proteins. Due to the lack of a specific antibody to mSAMD9L, a plasmid encoding a flag-epitope tagged mSAMD9L was used in transfection of 293 cells, which were subsequently infected with recombinant viruses expressing different V5-tagged viral proteins. Pulldown of VACV-K1, VACV-C7, MYXV-M62, SWPV-064 or YLDV-67 also precipitated mSAMD9L (Fig 3A). However, SPPV-063 failed to co-precipitate mSAMD9L. As expected, MYXV-M63 and MYXV-M64, which are two additional C7 homologs from MYXV previously known not to bind human SAMD9 [12], also failed to co-precipitate mSAMD9L.

Residue 134 and 135 of SPPV-063 were previously shown to be largely responsible for the defect of SPPV-063 in mouse cells, and substituting them with the corresponding ones found in SWPV-064 and VACV-C7 was sufficient to restore the host-range function in mouse cells without compromising the function in human cells [24]. The same substitution was found to result in the binding of SPPV-063 with mSAMD9L (Fig 3B). As controls, individual substitutions of two neighboring residues (residue 129 or 132) did not result in mSAMD9L binding, correlating with their lack of effect on the host-range function in mouse cells [24]. Interestingly, residues 134 and 135 are only adjacent to the three conserved loops of the C7-like proteins (Fig 3B) that are critical for the binding with SAMD9 [12].

### In diverse human cells, both the basal level of SAMD9 and interferon-induced SAMD9L could restrict vK1L^-^C7L^-^ replication

**Fig 4:**
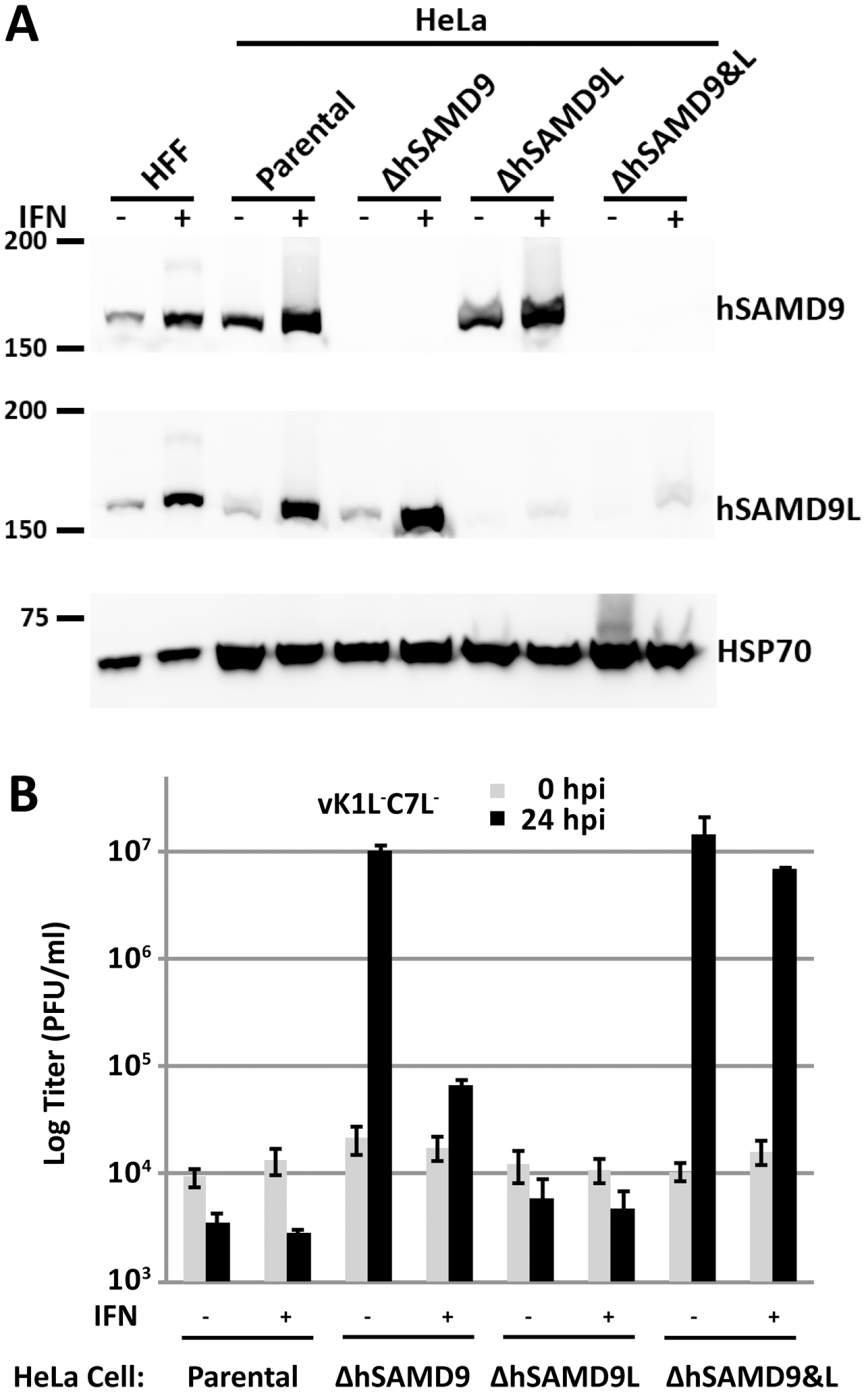
The basal level of hSAMD9 as well as interferon-induced hSAMD9L are both capable of restricting vK1L^-^C7L^-^ replication in HeLa cells. **(A)**. The validation of HeLa cell lines with knockout in hSAMD9 (ΔhSAMD9), hSAMD9L (ΔhSAMD9L) or both hSAMD9 and hSAMD9L (ΔhSAMD9&L) by Western blot. The clonally selected knockout cells were established with CRISPR-Cas9 technology. The cells were left untreated (-) or treated (+) with 200 U/ml of IFN-β for 24 hours. The levels of hSAMD9, hSAMD9L and HSP70 (loading control) proteins in the cell lysates were determined by Western blotting. Normal human foreskin fibroblasts (HFF) and the parental HeLa cells were used as controls. **(B)**. IFN-induced hSAMD9L restricts vK1L^-^C7L^-^ replication in ΔhSAMD9 cells. The cells were left untreated or treated with IFN-β as in *A* and infected with vK1L^-^C7L^-^. Viral growth was determined by measuring viral titers at 0 and 24 hpi.

The finding that mouse SAMD9L functions similarly to human SAMD9 as a poxvirus restriction factor raised the question about the function of human SAMD9L (hSAMD9L). Since knockdown of human SAMD9 (hSAMD9) was sufficient for abolishing the host restriction for vK1L^-^C7L^-^ in HeLa and A549 cells [14, 26], hSAMD9L was not previously suspected to play a role in host restriction. To find out whether hSAMD9L expression might be impaired in cell lines, we performed Western blot on HeLa cells and normal human fibroblasts derived from foreskin, and found that both hSAMD9 and hSAMD9L were constitutively expressed and their expression was further induced by IFN-β (Fig 4A).

To study hSAMD9L function independent of hSAMD9, we knocked out hSAMD9 from HeLa cells with CRISPR-Cas9. In a HeLa cell clone with hSAMD9 knockout (ΔhSAMD9), no hSAMD9 was detected by Western blot even after the cells were treated with IFN-β (Fig 4A). ΔhSAMD9 cells were fully permissive for the replication of vK1L^-^C7L^-^, as reported previously [14]. However, pretreating the ΔhSAMD9 cells with IFN-β restored the host restriction for vK1L^-^C7L^-^, reducing the viral yield by more than 100-fold compared to that in untreated cells (Fig 4B). To test whether hSAMD9L was responsible for the restriction, we knocked out hSAMD9L from ΔhSAMD9 and the parental HeLa cells. Both the hSAMD9L single-knockout cells (ΔhSAMD9L) or hSAMD9 and hSAMD9L double knockout cells (ΔhSAMD9&L) had no detectable level of hSAMD9L protein in untreated cells and only a trace amount of the protein after IFN-β stimulation (Fig 4A). In contrast to ΔhSAMD9 cells, ΔhSAMD9&L cells remained permissive for vK1L^-^C7L^-^ after IFN-β treatment (Fig 4B), and the growth of vK1L^-^C7L^-^ was similar to that of the wild type (WT) VACV WR strain (S3 Fig), indicating that IFN-β-induced hSAMD9L restricted the replication of vK1L^-^C7L^-^ in the absence of hSAMD9. ΔhSAMD9L cells were similar to the parental HeLa cells in restriction of vK1L^-^C7L^-^ (Fig 4B).

To ensure that the HeLa cell results are representative of the phenotype in human cells, we performed similar knockout studies in a variety of human cells that we identified to be restrictive of the replication of vK1L^-^C7L^-^. These include normal human foreskin fibroblasts (HFFs) and cancer cells derived from skin (A431), breast (HS578T and MDA-MB-231), cervix (HT-3), prostate (PC-3) and ovary (SKOV3). We transduced these cells with a lentivirus expressing a gRNA targeting either hSAMD9 or hSAMD9L and pooled the stably transduced cells. The SAMD9 and SAMD9L protein level in the pooled cells was reduced but not eliminated as in the clonally selected HeLa knockout cells (S4A & S5A Figs), presumably because the targeted gene was repaired with in-frame indels in a fraction of the cells. Thus, the cells were conservatively speaking gene knockdown instead of knockout cells. Nevertheless, the results were similar to that in knockout HeLa cells. The HFFs were less stringent than the HeLa cells in restricting vK1L^-^C7L^-^, allowing the viral titer increase by ~3-fold after 24 hours of infection (S4B Fig). The knockdown of hSAMD9 or hSAMD9L from HFFs increased the viral yield by ~110-fold or ~6-fold, respectively, indicating that hSAMD9 is the dominant restriction factor in HFFs. Correspondingly, robust viral late protein synthesis was only observed in hSAMD9-knockdown HFFs (S4A Fig). IFN treatment of hSAMD9-knockdown HFF cells restored the restriction for vK1L^-^C7L^-^ (S4B Fig). The results from the knockout studies in all the cancer cell lines were also similar to that in HeLa cells in that the knockdown of hSAMD9, but not hSAMD9L, abolished the host restriction for vK1L^-^C7L^-^ (S5B Fig). Moreover, IFN treatment of hSAMD9-knockdown cells restored the restriction for vK1L^-^C7L^-^ (S5C Fig).

Thus, in all human cells that we have tested, the basal level of hSAMD9 as well as IFN-induced hSAMD9L are both capable of restricting vK1L^-^C7L^-^ replication.

### VACV K1, C7 and C7 homologs from diverse poxviruses could overcome hSAMD9L restriction, but the sheeppox virus C7 homolog has reduced potency due to the variation of two residues

**Fig 5:**
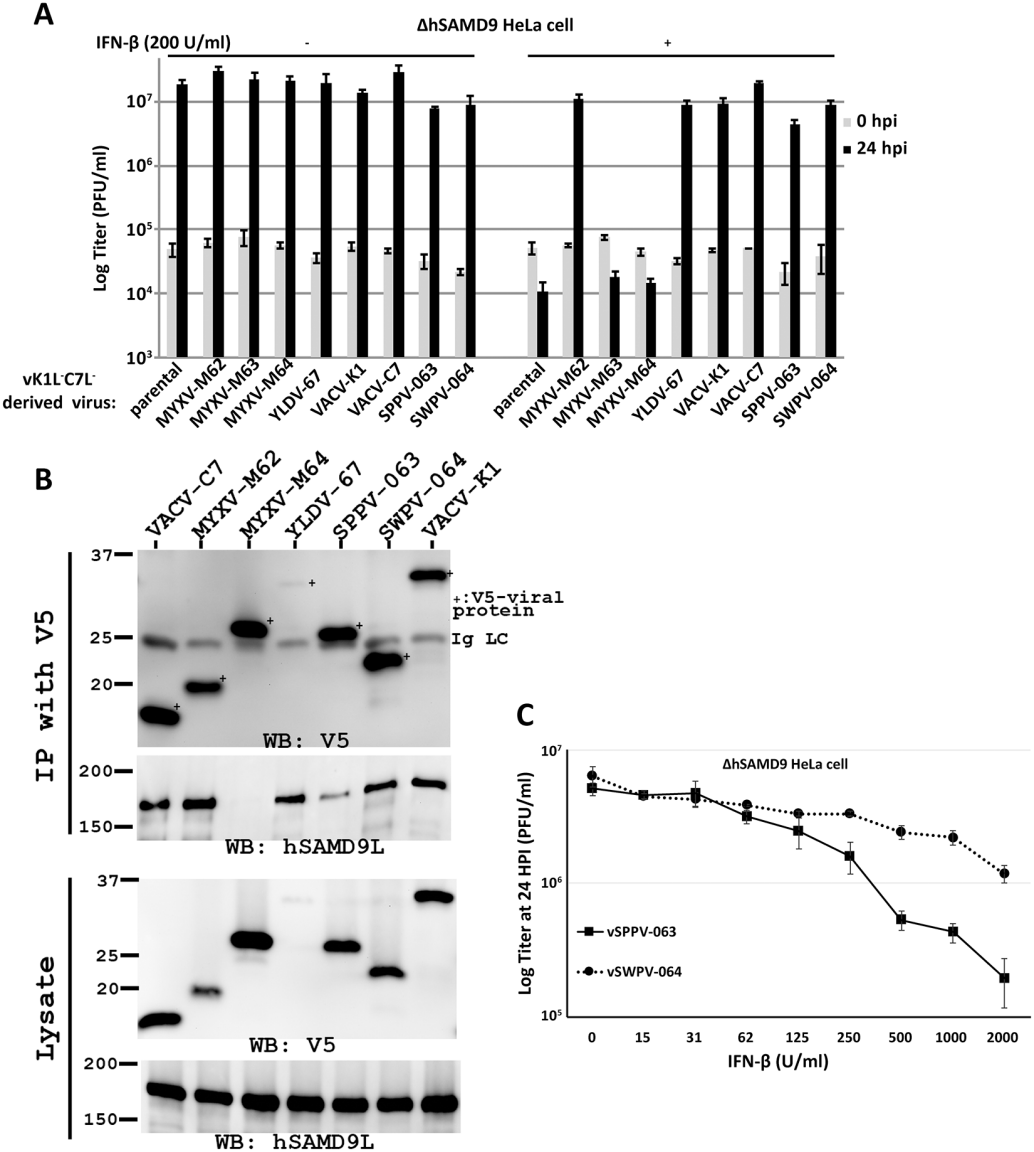
VACV K1, C7 and C7 homologs from diverse poxviruses could inhibit hSAMD9L, but the C7 homolog from sheeppox virus has a reduced potency. **(A)**. VACV K1, C7 and C7 homologs from diverse poxviruses could overcome restriction by hSAMD9L. ΔhSAMD9 HeLa cells were left untreated (-) or treated (+) with 200 U/ml of IFN-β for 24 hours, before they were infected with the panel of vK1L^-^C7L^-^-derived VACVs expressing VACV-K1 or a C7 homolog from different poxviruses (indicated). Viral growth was determined as described before. **(B)**. The sheeppox virus C7 homolog (SPPV-063) has a reduced affinity to hSAMD9L. The ΔhSAMD9 HeLa cells were treated with IFN-β and then lysed for immunoprecipitation with an anti-V5 antibody. hSAMD9L and the V5-tagged viral proteins in the cell lysate and precipitate were detected respectively with hSAMD9L and V5 antibody in Western blot. **(C)**. SPPV-063 has a reduced potency in inhibiting hSAMD9L. The ΔhSAMD9 HeLa cells were treated with the indicated concentration of IFN-β (from 0 to 2000 U/ml) for 24 hours, before they were infected with vK1L^-^C7L^-^-derived VACVs expressing either SPPV-063 or SWPV-064. Viral titers at 24 hpi were plotted against the concentrations of IFN-β used for the induction of hSAMD9L.

To find out whether mammalian poxviruses could overcome the restriction of hSAMD9L, we induced hSAMD9L expression in ΔhSAMD9 HeLa cells with 200 U/ml of IFN-β and then infected the cells with our panel of vK1L^-^C7L^-^-derived VACVs. Viruses that expressed VACV-K1, VACV-C7, MYXV-M62, SPPV-063, SWPV-064 and YLDV-67 (but not MYXV-M63 and MYXV-M64) grew in IFN-treated ΔhSAMD9 cells (Fig 5A), indicating that all known SAMD9 antagonists could also antagonize hSAMD9L. We then studied these viral proteins for binding of hSAMD9L. While MYXV-M63 (S6 Fig) and MYXV-M64 did not co-precipitate hSAMD9L, VACV-K1, VACV-C7, MYXV-M62, YLDV-67, SPPV-063 and SWPV-064 co-precipitated hSAMD9L (Fig 5B). Among them, SPPV-063 precipitated a lower amount of hSAMD9L, indicating a reduced affinity. This defect was again due to residue 134 and 135 of SPPV-063, as substitution of these two residues increased the precipitation of hSAMD9L without affecting the precipitation of hSAMD9 (S6 Fig).

To determine whether the weaker hSAMD9L binding affinity by SPPV-063 resulted in reduced inhibitory potency, we induced an increasingly higher level of hSAMD9L from ΔhSAMD9 HeLa cells with IFN-β and then infected the cells with either vSPPV-063 or vSWPV-064. The two viruses grew equally well in untreated ΔhSAMD9 cells, reaching similar titers after 24 hours of infection (Fig 5C). The viral yields were gradually reduced by the increasing concentrations of IFN-β, and the magnitudes of the reduction were significantly larger for vSPPV-063 than for vSWPV-064 when the interferon concentration was greater than 250 U/ml, indicating that SPPV-063 is less effective than SWPV-064 at antagonizing hSAMD9L.

## Discussion

SAMD9 was recently identified as a restriction factor for poxviruses in human cell lines [11, 14]. However, whether SAMD9 is important for host defense against poxvirus pathogenesis and whether similar antiviral defense exists in other mammals, especially those that lack a SAMD9 ortholog, were not established. In this study, we showed that the SAMD9 paralog, SAMD9L, from mammalian species as diverse as mice and humans, functions similarly to human SAMD9 as a poxvirus restriction factor. Since at least one of the two paralogs is present in all mammals with a completely sequenced genome [20], our finding indicates that SAMD9 and/or SAMD9L-mediated antiviral defense is conserved in mammals. This conservation allowed us to use the mouse model to reveal the critical role of SAMD9/L in host defense against poxvirus pathogenesis. Studies of SAMD9/L from two different mammalian species and the SAMD9/L inhibitors from diverse mammalian poxviruses revealed some host species-specific difference in SAMD9/L, which could serve as a barrier for cross-species poxvirus infection. This knowledge could be useful in assessing the potential of a given poxvirus in switching or expanding its host range.

Mouse is one of the mammalian species that have lost SAMD9 but maintained SAMD9L. Mouse SAMD9L is 53% and 71% identical to human SAMD9 and SAMD9L at amino acid level, respectively. Through gene knockout with both the conventional technique as well as the CRISPR-Cas9 technique, we found that mouse SAMD9L was essential for restricting the replication of a model poxvirus, a vaccinia virus mutant deleted of both K1L and C7L (vK1L^-^C7L^-^). 3T3 cells with CRISPR knockout of *SAMD9L* as well as *SAMD9L*^*-/-*^ MEFs were permissive for the replication of vK1L^-^C7L^-^. Furthermore, vK1L^-^C7L^-^ caused lethal intranasal infection only in *SAMD9L*^-/-^ mice but was completely avirulent in *SAMD9L*^+/+^ and *SAMD9L*^+/-^ mice, demonstrating the potency of SAMD9L as a restriction factor at the organismal level.

Human is one of the mammalian species that have both SAMD9 and SAMD9L, located head-to-tail in adjacent positions of the same chromosome. Previous phylogenetic studies suggested that SAMD9 and SAMD9L originated from a common ancestor through an ancient gene duplication event [20]. Gene duplications have a major role in evolution of new biological functions [27]. With about 60% amino acid sequence identity, human SAMD9 and SAMD9L could have diverged to take on different functions. This idea would be consistent with the previous findings that loss-of-function mutations in only SAMD9 cause NFTC in humans [15] and that knockdown of SAMD9 was sufficient for abolishing the restriction for vK1L^-^C7L^-^ in several human cell lines [14, 26]. We were thus initially surprised to find that human SAMD9L functioned similarly to SAMD9 as a restriction factor for poxviruses. The advent of CRISPR-Cas9 genome editing technique made it possible to readily knock out one or both of the human paralogs from various human cells. The knockouts in tumor cell lines from various tissues and the normal human foreskin fibroblasts showed that both SAMD9 and SAMD9L, when sufficiently expressed, could inhibit vK1L^-^C7L^-^ replication. In many human cells, the basal level of SAMD9 is sufficient for restricting vK1L^-^C7L^-^, but SAMD9L has to be induced by IFN to have the same effect. This probably only reflects a difference in gene regulation of the two paralogs in human cells but not any real difference in their potency or mechanism of action. Mouse SAMD9L is also an interferon-stimulated gene [28], but the basal level of SAMD9L in mouse cells is sufficient for restricting vK1L^-^C7L^-^, again suggesting that the role of IFN in SAMD9L function is merely to induce SAMD9L to a sufficient level in some cell types. Similarly, the only contribution made by K1 and C7 in vaccinia virus antagonism of IFN or IRF1 [24, 25] is likely the inhibition of IFN- or IRF1-induced SAMD9/L, as vK1L^-^C7L^-^ was not sensitive to IFN in cells that had SAMD9/L knockout.

Both human SAMD9 and SAMD9L have strong anti-proliferative function, and gain-of-function (GoF) mutations of SAMD9 or SAMD9L cause multisystem developmental disorder. GoF SAMD9 mutations cause MIRAGE (myelodysplasia, infection, restriction of growth, adrenal hypoplasia, genital phenotypes, and enteropathy) disorder [29], while GoF SAMD9L mutations cause a similar disorder characterized with cytopenia, immunodeficiency, and neurological symptoms [19, 30]. In both cases, the GoF mutations predispose to myelodysplastic syndrome by facilitating the selection for a loss of the chromosome that contain the mutations [19, 30]. So why are both *SAMD9* and *SAMD9L* kept in humans and many other mammals? We can only postulate that the duplication of SAMD9 gene and the divergence of sequence and expression pattern represent a strategy for retaining two drastically different “alleles” of SAMD9/L in the same host without the deleterious effect of both genes being constitutively active. Having two different alleles of SAMD9/L would have given the hosts more flexibility to rapidly evolve SAMD9/L to evade the viral inhibitors, perhaps during a time of mammalian evolution when the challenge from poxviruses was particularly rampant. Phylogenetic analysis has showed that both SAMD9 and SAMD9L genes have been subjected to sustained positive selection [20].

**Fig 6:**
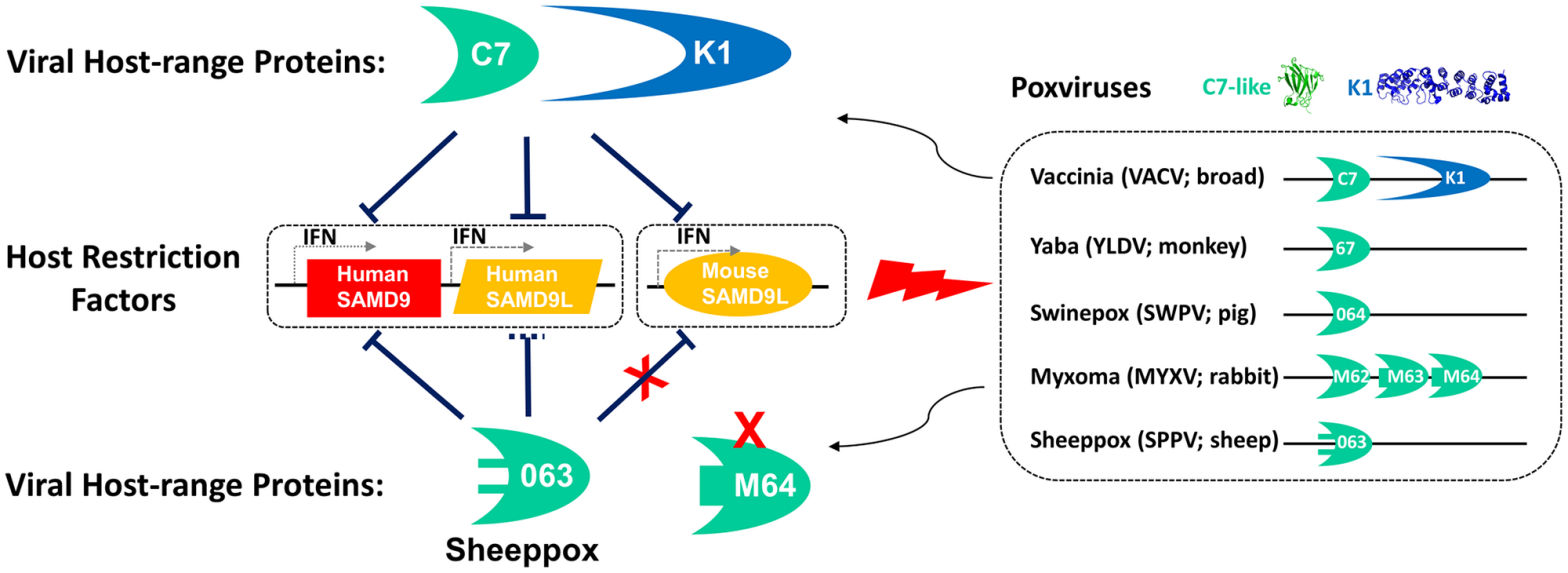
Summary of SAMD9/L restriction factors in human and mice and their antagonism by different poxviruses. The K1 and C7-like proteins encoded by the representative mammalian poxviruses are shown on the right. The two amino acid variations in SPPV-063 is shown as two additional lines.

Corresponding to the conservation of SAMD9/L in mammals, the distribution of SAMD9/SAMD9L inhibitors in mammalian poxviruses are also very broad. Nearly all mammalian poxviruses encode a C7 homolog that provides a host-range function similar to VACV C7 [10, 24, 31], and a few poxviruses also encode K1 (Fig 6). All the functional C7 homologs and K1 were previously shown to bind and inhibit human SAMD9 [11, 12, 14]. The breadth of their antagonistic activities is now expanded to include human and mouse SAMD9L. Among the C7 homologs from a wide variety of different mammalian poxvirus, the one from sheeppox virus, SPPV-063, stands out as the only one that displays a specificity for SAMD9. SPPV-063 could bind and inhibit human SAMD9, but it has reduced potency against human SAMD9L and failed to inhibit mouse SAMD9L. Remarkably, its binding to mouse SAMD9L could be restored simply by substituting two residues, suggesting that subtle difference between SAMD9 and SAMD9L might be responsible for their different ability in resisting SPPV-63. Interesting, these two residues are only adjacent to the “three-fingered molecular claw” that is critical for SAMD9 binding [12], indicating that they may modulate the conformation of “the claw” to influence its binding specificity. Sheeppox virus, goatpox virus, and lumpy skin disease virus (LSDV) are members of the capripoxvirus genus with a narrow host-range in ruminants. All capripoxviruses encode a nearly identical C7 homolog (>97% identical) as SPPV-063, with the same residues at position 134 and 135, suggesting that they all preferentially antagonize SAMD9. This correlates with the loss of SAMD9L in ruminants. Thus, one evolutionary scenario is that the ancestor of SPPV-063 could tolerate genetic drift that only disrupted SAMD9L binding, after the ancestor of extant capripoxviruses established a niche host in ruminants and was no longer restricted by SAMD9L. While the resulted failure of capripoxviruses in antagonizing mouse SAMD9L could account for the host restriction of capripoxviruses in mice, the resulted reduction in potency against interferon-induced human SAMD9L could only partly explain the host restriction of capripoxviruses in humans. We suspect that similar genetic drift might have occurred to other capripoxvirus host-range genes, resulting in their specialization to the ruminant hosts and altogether contributing to the host restriction of capripoxviruses in non-ruminant hosts. This idea is supported by a previous report that the sheeppox virus homolog of VACV host range gene E3 failed to provide the host range function in HeLa cells [32]. That mouse SAMD9L is completely resistant to SPPV-063 while human SAMD9L is only partially resistant could have been the result of a stronger positive selection in rodents, perhaps by some poxviruses that have similar inhibitory profile as the capripoxviruses. Overall, our studies suggest that host species-specific difference in SAMD9/L gene repertoire contributes to the barrier for cross-species poxvirus transmission.

## Materials and methods

### Ethics statement

The animal studies reported in this paper were approved by the Institutional Animal Care and Use Committee at the University of Texas Health Science Center at San Antonio (Protocol no. 14040x), and adhere to the guidelines and recommendations from the Guide for the Care and Use of Laboratory Animals of the National Institutes of Health and the Office of Laboratory Animal Welfare. The Institution has an Animal Welfare Assurance on file with the NIH Office of Laboratory Animal Welfare (assurance number A3345-01). The care and use of animals is in accordance with the NRC Publication, as revised in 2011, “Guide for the Care and Use of Laboratory Animals,” and other applicable Federal regulations.

### Cells

VERO (ATCC CCL-81), HeLa 229 (ATCC CCL-2.1), NIH/3T3 (ATCC CRL-1658), A431 (ATCC CRL-1555), HS578T (ATCC HTB-126), HT-3(ATCC HTB-32), MDA-MB-231 (ATCC HTB-26), SKOV3 (ATCC HTB-77), and PC-3 (ATCC CRL-1435) were originally from ATCC. HEK 293FT was from Thermo Fisher Scientific (cat. no. R70007). Mouse embryonic fibroblasts (MEFs) were generated from embryos of SAMD9L^−/−^ and SAMD9L^+/+^ mice according to standard protocols. Human foreskin fibroblasts (HFFs), described in [33], were kindly provided by Dr. Zhilong Yang.

### Viruses

WT VACV WR strain, K1L and C7L deletion VACV (vK1L^-^C7L^-^) and a panel of vK1L^-^C7L^-^-derived recombinant viruses expressing VACV-K1 (vVACV-K1L) or a C7 homolog from different poxviruses (vVACV-C7L, vYLDV-67R, vMYXV-M62R, vMYXV-M63R, vMYXV-M64R, vSPPV-063 and vSWPV-064) were described before [10, 25, 34]. vSPPV-063 with mutations at residue 129,132, 134&135 of SPPV-063 were also described before [24]. All viruses were propagated and titrated on VERO cells.

### Generation of cells with gene knockouts with CRISPR-Cas9 technology

The plasmids used for the gene knockout were constructed from lentiCRISPRv2 (a gift from Feng Zhang, Addgene plasmid #52961) according to the published protocol [35]. In brief, lentiCRISPRv2 was digested with *BsmBI* and ligated with a pair of oligonucleotides with the specific guide sequence. For each target gene, two guide sequences were chosen from the human or mouse genome-wide sgRNA library [36]. They were as follows: 5’-TATCCGGGAACCACGGTTCG-3’ (mSAMD9L #1), 5’-ACACCAACAATTTCCCCGTG-3’ (mSAMD9L #2), 5’-TTGACTGAACAAGACGTGAA-3’ (hSAMD9 #1), 5’-GAGAATTTGTTCTTCGATAC-3’ (hSAMD9 #2), 5’- CCTGACCAGTTAGACGACGC-3’ (hSAMD9L #1), and 5’-GGCTAGCTCTAGGGATATCC-3’ (hSAMD9L #2).

The lentiCRISPRv2-derived plasmid was either transfected directly to the target cells or used in making lentiviruses for transduction of the target cells. In the latter case, the lentiviral plasmid and the packaging plasmids pMD2.G and psPAX2 (gifts from Didier Trono, Addgene plasmid # 12259 & 12260) were transfected into HEK 293FT cells with lipofectamin 3000 (Thermo Fisher Scientific). 60 hr post transfection, culture supernatants were collected, clarified by centrifugation, passed through a 0.45 μm filter, and used for transduction. For lentiviral transduction, the lentiviruses and the target cells in medium containing 10 μg/ml polybrene were centrifuged at 1,500 rpm for 2 hr. 24 hr after either transfection or transduction, the cells were subjected to puromycin selection for 2 (transfection method) or 7 (transduction method) days. The puromycin concentrations are 3 μg/ml for HeLa, 10 μg/ml for 3T3, and 2 μg/ml for all other cells.

For each gene knockout in 3T3 and HeLa cells, two separate knockouts with different guides were performed. Clones of the knockout cells were isolated and validated by Western blotting for hSAMD9 or hSAMD9L or genotyping for mSAMD9L. For genotyping, the genomic DNA of the clones was extracted using the QuickExtract DNA extraction kit (Epicentre). ~500 bp of DNA flanking the target site was PCR-amplified with the primer pair (5’-GGCCACTCAATCTCATTGACCCAAT-3’ and 5’-TGCCCAGGATATTCTTAGAGCTAGC-3’) and cloned to pGEM vector (Promega). The sequence of 10–20 pGEM clones was determined by Sanger sequencing.

For knockout of hSAMD9 or hSAMD9L in additional human cell lines and HFFs, only guide #1 described above was used for the knockout. The stably transduced target cells were pooled without clonal selection, and the knockout was validated by Western blot.

### Intranasal infection of mice with VACV

Mice with different SAMD9L genotypes used in the infection experiment were generated from breeding pairs of *SAMD9L*^*+/-*^ mice [21]. At around 4 to 5 weeks old, the mice were infected intranasally with viruses in 20 μl PBS as described previously [10]. The viruses used were purified through a sucrose cushion sedimentation according to the standard protocol [37]. Individual mice were weighed every day and euthanized when 25% of the body weight was lost. Statistical comparisons of body weight changes among groups were analyzed by two way ANOVA using GraphPad Prism 5.0. Values of p < 0.05 were considered statistically significant. The anti-VACV serum antibody titers of some infected mice were determined by ELISA with purified VACV virions as described previously [38]. All mouse protocols were approved by UTHSCSA IACUC.

### mSAMD9L expression plasmid

mSAMD9L ORF was PCR-amplified with the primer pair (5’-AGTGGACAAGTAACTCAACCAAAATTG-3’ and 5’-GATCACTTTTATGCCATATGCC-3’) from cDNA synthesized from 3T3 cellular mRNAs. A 3XFlag tag sequence and a HA tag sequence were then appended to the 5’ and 3’ end of the mSAMD9L ORF by recombinant PCR, and the final PCR product was inserted between KpnI and SacII sites of the pcDNA3.1/V5-His-topo vector (Thermo Fisher Scientific). The mSAMD9L ORF was completely sequenced and found to have one amino acid difference (Val instead of Ile at 459) compared to the mSAMD9L reference sequence in GenBank (NP_034286.2). The difference was not corrected, as SAMD9L from most rodents also has Val at this position.

### Viral growth analysis

Cells in 12-well plates were incubated with 1 PFU per cell of different viruses for 2 h at room temperature. Following adsorption, the cells were washed twice with phosphate-buffered saline (PBS). One set of the cells was harvested immediately as the 0 hr post infection sample, while the other set were moved to 37°C incubator to initiate viral entry and harvested at 24 hr post infection. The viral titers in the cell lysates were determined by plaque assays on VERO cells. For testing the effect of IFN, human or murine cells were treated with 200 U/ml of human or murine IFN-β (PBL Biomedical Laboratories) for 24 hr, before the cells were infected with VACV as described above. For assessing viral late protein expression, Western blot with a mAb against the VACV late protein WR148 (clone HE7) [39] was performed.

### Immunoprecipitation and western blot analysis

For immunoprecipitation of hSAMD9 or hSAMD9L, HeLa cells or IFN-treated ΔhSAMD9 HeLa cells were infected with different VACV viruses. For immunoprecipitation of mSAMD9L, 293FT cells were transfected with the mSAMD9L expression plasmid and then infected with different VACVs at 48 hr post transfection. After 8 hr of infection, the cells were lysed on ice with a lysis buffer (0.1% (w/v) NP-40, 50 mM Tris, pH 7.4, 150 mM NaCl) supplemented with protease inhibitor cocktail tablets (Roche Molecular Biochemicals). The cleared cell lysates were mixed with 50 μl of 50% (vol/vol) V5-agarose beads (Sigma-Aldrich) for 30 min at 4°C. After washing with lysis buffer, the beads were resuspended in SDS sample buffer, the eluted proteins were resolved by SDS-PAGE and detected with Western blot as described previously [34]. The detection antibodies were mouse monoclonal antibodies (mAb) against V5 (Sigma-Aldrich; clone V5-10), Flag tag (Sigma-Aldrich) or HSP70 (Santa Cruz), and rabbit polyclonal antibodies against hSAMD9 (Sigma-Aldrich, HPA-21319) and hSAMD9L (Proteintech, 25173-1-AP).

## Supporting information

S1 Fig Related to Fig 1A. **(A)**. Genotyping ΔmSAMD9L 3T3 cells. The mSAMD9L knockout cell lines (ΔmSAMD9L) were constructed by transient transfection of 3T3 cells with a plasmid encoding Cas9 and a gRNA targeting mSAMD9L (guide#1 or guide#2). The targeted SAMD9L sequence is underlined with the PAM sequence in bold italics and the encoded amino acid sequence shown above the DNA sequence. Shown below the target are the genomic sequences from representative cell clones (ΔmSAMD9L#1 and ΔmSAMD9L#2). Gray and crossed-out sequence indicates deletion. ^ indicates insertion. The number after the + and − denotes the number of indels, and the number before the “x” denotes the number of times the sequence was detected from a total of 10–20 cloned PCR products. **(B)**. The restriction of K1L and C7L deletion vaccinia virus (vK1L^-^C7L^-^) in 3T3 cells was abolished by knocking out mSAMD9L with CRISPR-Cas9. A validated mSAMD9L knockout cell clone (ΔmSAMD9L #2) and the parental 3T3 cells were infected with vK1L^-^C7L^-^ at an MOI of 1 PFU/cell. Viral growth was determined by measuring viral titers at 0 and 24 hour-post-infection (hpi).
(PDF)

S2 Fig **(A)**. vK1L^-^C7L^-^ infection induced anti-VACV antibody response in mice. Mice described in Fig 2A were euthanized, and their anti-VACV serum antibody titer was determined by ELISA against purified VACV. **(B)**. Mice described in Fig 2C were euthanized at day 5 post infection and their lungs and spleen were harvested. Approximately 16 mg of spleen and 5 mg of lung from each mouse were homogenized and their viral loads were determined by plaque assay on VERO cells.
(PDF)

S3 Fig WT VACV WR can overcome the restriction by both hSAMD9 and hSAMD9L. Related to Fig 4B. The cells were treated as in Fig 4 and infected with WT VACV WR. Viral growth was determined by measuring viral titers at 0 and 24 hpi.
(PDF)

S4 Fig The basal level of hSAMD9 as well as interferon-induced hSAMD9L are both capable of restricting vK1L^-^C7L^-^ in normal human foreskin fibroblasts. **(A)**. Knockdown of hSAMD9 but not hSAMD9L in human foreskin fibroblasts (HFFs) rescued viral late protein expression by vK1L^-^C7L^-^. HFFs were transduced with a lentivirus expressing a gRNA targeting either hSAMD9 or hSAMD9L, and stably transduced cells were pooled. The knockdown of hSAMD9 or hSAMD9L was confirmed by Western blot (left). A set of the cells were infected with vK1L^-^C7L^-^ and the level of a representative VACV late protein WR148 was determined by Western blot (right). Par., parental; Δ9, hSAMD9-knockdown; Δ9L, hSAMD9L-knockdown. **(B)**. IFN restored host restriction for vK1L^-^C7L^-^ in hSAMD9-knockdown HFFs. The parental and the knockdown cells were left untreated or treated with IFN-β and infected with vK1L^-^C7L^-^. Viral growth was determined by measuring viral titers at 0 and 24 hpi.
(PDF)

S5 Fig The basal level of hSAMD9 as well as interferon-induced hSAMD9L are both capable of restricting vK1L^-^C7L^-^ in human cell lines derived from diverse tissues. **(A)**. Either hSAMD9 or hSAMD9L was knocked down from various human cells with CRISPR-Cas9 as described in S4 Fig. Pooled knockdown cells were used without clonal selection, and the knockdown was validated by Western blot. Par., parental; Δ9, hSAMD9-knockdown; Δ9L, hSAMD9L-knockdown. **(B)**. Knockdown of hSAMD9 but not hSAMD9L abolished the restriction for vK1L^-^C7L^-^ in human cells from diverse tissues. The parental and the knockdown cells were infected with vK1L^-^C7L^-^ at an MOI of 1 PFU/cell. **(C)**. IFN restored host restriction for vK1L^-^C7L^-^ in hSAMD9-knockdown (ΔhSAMD9) cells. Various ΔhSAMD9 cells were left untreated or treated with IFN-β and infected with vK1L^-^C7L^-^. Viral growth was determined by measuring viral titers at 0 and 24 hpi.
(PDF)

S6 Fig Substitution of residue 134 and 134 of SPPV-063 increases binding to hSAMD9L without any adverse effect on binding to hSAMD9. IFN-treated ΔSAMD9 HeLa cells (A) or untreated parental HeLa cells (B) were infected with vK1L^-^C7L^-^-derived virus that expressed MYXV-M63 (negative control) or SPPV-063 (WT or mutated). The C7 homolog was precipitated with an anti-V5 antibody, and the co-precipitated hSAMD9 or hSAMD9L was detected by Western blot.
(PDF)
