# Supplementary material for: A paralogous pair of mammalian host restriction factors form a critical host barrier against poxvirus infection
Source: PLoS Pathog. 2018 Feb 15;14(2):e1006884. doi: 10.1371/journal.ppat.1006884 (PMC5831749; doi:10.1371/journal.ppat.1006884)
Supplement: S1 Fig — (A). Genotyping ΔmSAMD9L 3T3 cells. The mSAMD9L knockout cell lines (ΔmSAMD9L) were constructed by transient transfection of 3T3 cells with a plasmid encoding Cas9 and a gRNA targeting mSAMD9L (guide#1 or guide#2). The targeted SAMD9L sequence is underlined with the PAM sequence in bold italics and the encoded amino acid sequence shown above the DNA sequence. Shown below the target are the genomic sequences from representative cell clones (ΔmSAMD9L#1 and ΔmSAMD9L#2). Gray and crossed-out sequence indicates deletion. ^ indicates insertion. The number after the + and − denotes the number of indels, and the number before the “x” denotes the number of times the sequence was detected from a total of 10–20 cloned PCR products. (B). The restriction of K1L and C7L deletion vaccinia virus (vK1L-C7L-) in 3T3 cells was abolished by knocking out mSAMD9L with CRISPR-Cas9. A validated mSAMD9L knockout cell clone (ΔmSAMD9L #2) and the parental 3T3 cells were infected with vK1L-C7L- at an MOI of 1 PFU/cell. Viral growth was determined by measuring viral titers at 0 and 24 hour-post-infection (hpi). (PDF) [file ppat.1006884.s001.pdf]

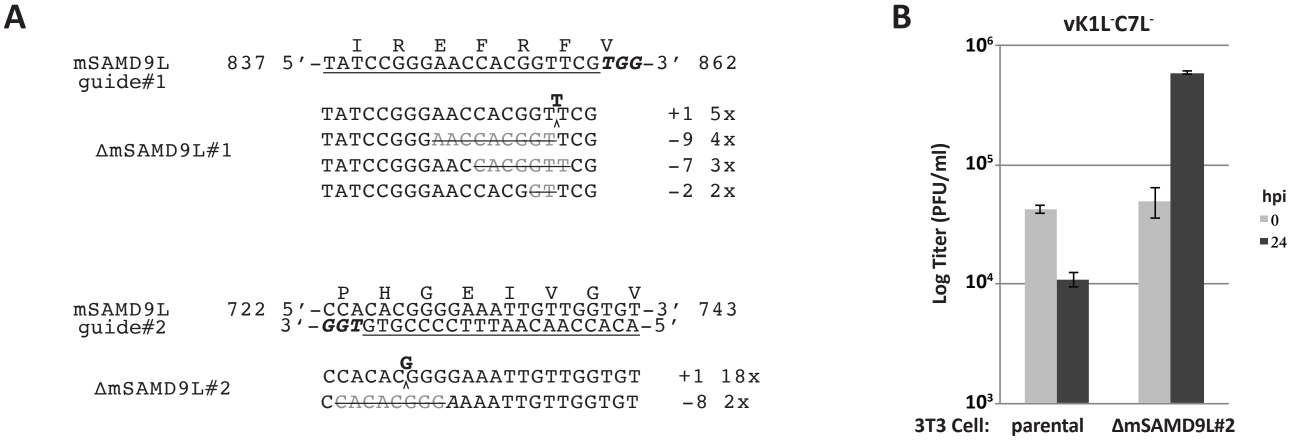
A
I R E F R F V
mSAMD9L guide#1 837 5′-TATCCGGGAACCACGGTTCGTGG-3′ 862
ΔmSAMD9L#1
TATCCGGGAACCACGGTTTCG +1 5x
TATCCGGGAACCACGGTTCG -9 4x
TATCCGGGAACCACGGTTCG -7 3x
TATCCGGGAACCACGGTTCG -2 2x
P H G E I V G V
mSAMD9L guide#2 722 5′-CCACACGGGGAAATTGTTGGTGT-3′ 743
3′-GGTGTGCCCCTTTAACAACCACA-5′
ΔmSAMD9L#2
CCACACGGGGGAAATTGTTGGTGT +1 18x
CCACACGGGAAAATTGTTGGTGT -8 2x
B
vK1L-C7L-
Log Titer (PFU/ml)
hpi
0
24
3T3 Cell:
parental
ΔmSAMD9L#2
