# Supplementary material for: A paralogous pair of mammalian host restriction factors form a critical host barrier against poxvirus infection
Source: PLoS Pathog. 2018 Feb 15;14(2):e1006884. doi: 10.1371/journal.ppat.1006884 (PMC5831749; doi:10.1371/journal.ppat.1006884)
Supplement: S2 Fig — (A). vK1L-C7L- infection induced anti-VACV antibody response in mice. Mice described in Fig 2A were euthanized, and their anti-VACV serum antibody titer was determined by ELISA against purified VACV. (B). Mice described in Fig 2C were euthanized at day 5 post infection and their lungs and spleen were harvested. Approximately 16 mg of spleen and 5 mg of lung from each mouse were homogenized and their viral loads were determined by plaque assay on VERO cells. (PDF) [file ppat.1006884.s002.pdf]

A

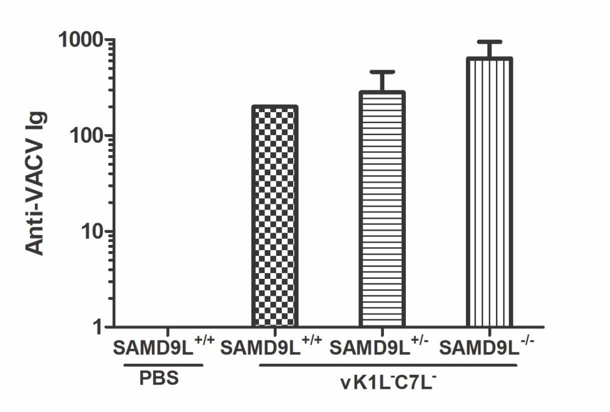

B

**Virus**	**Mouse**	**Viral titer in Lung (PFU/5 mg tissue)**	**Viral titer in spleen (PFU/16 mg tissue)**
WR	SAMD9L$^{-/-}$ #1	1.9×10^{7}	1.9×10^{3}
WR	SAMD9L$^{-/-}$ #2	2.5×10^{7}	2.8×10^{3}
vK1L^{-}C7L^{-}	SAMD9L$^{+/+}$ all	below detection (<10)	below detection (<10)
vK1L^{-}C7L^{-}	SAMD9L$^{+/-}$ all	below detection (<10)	below detection (<10)
