# Supplementary figures and images for: A paralogous pair of mammalian host restriction factors form a critical host barrier against poxvirus infection
Source: PLoS Pathog. 2018 Feb 15;14(2):e1006884. doi: 10.1371/journal.ppat.1006884 (PMC5831749; doi:10.1371/journal.ppat.1006884)

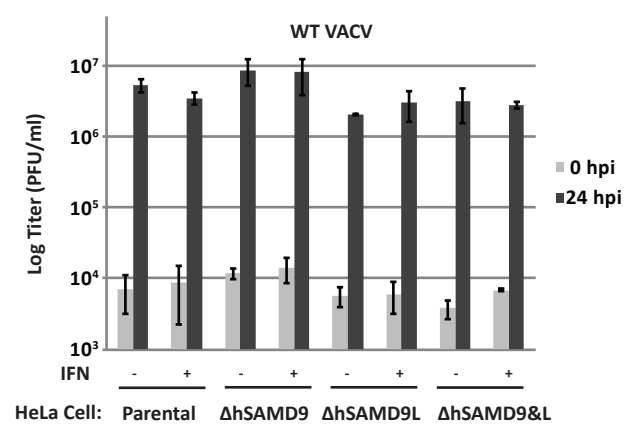

Supplement: S3 Fig — Related to Fig 4B. The cells were treated as in Fig 4 and infected with WT VACV WR. Viral growth was determined by measuring viral titers at 0 and 24 hpi. (PDF) [file ppat.1006884.s003.pdf]

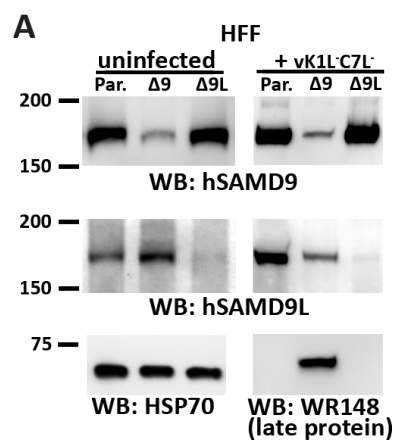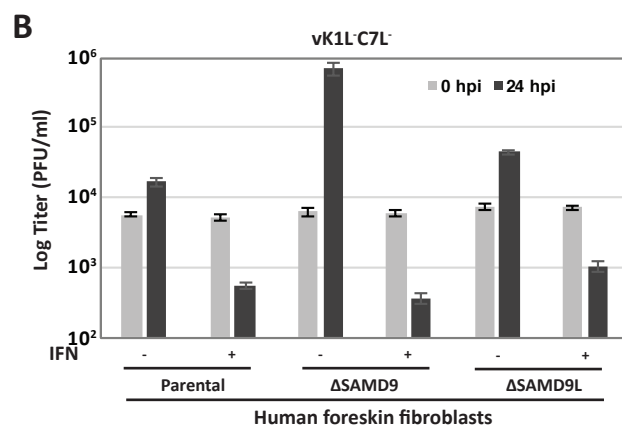

Supplement: S4 Fig — (A). Knockdown of hSAMD9 but not hSAMD9L in human foreskin fibroblasts (HFFs) rescued viral late protein expression by vK1L-C7L-. HFFs were transduced with a lentivirus expressing a gRNA targeting either hSAMD9 or hSAMD9L, and stably transduced cells were pooled. The knockdown of hSAMD9 or hSAMD9L was confirmed by Western blot (left). A set of the cells were infected with vK1L-C7L- and the level of a representative VACV late protein WR148 was determined by Western blot (right). Par., parental; Δ9, hSAMD9-knockdown; Δ9L, hSAMD9L-knockdown. (B). IFN restored host restriction for vK1L-C7L- in hSAMD9-knockdown HFFs. The parental and the knockdown cells were left untreated or treated with IFN-β and infected with vK1L-C7L-. Viral growth was determined by measuring viral titers at 0 and 24 hpi. (PDF) [file ppat.1006884.s004.pdf]

**A**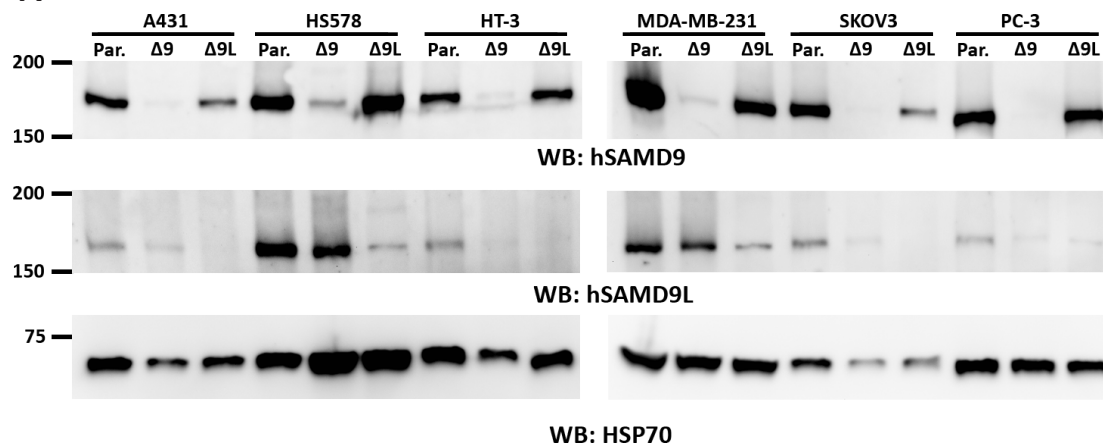**B**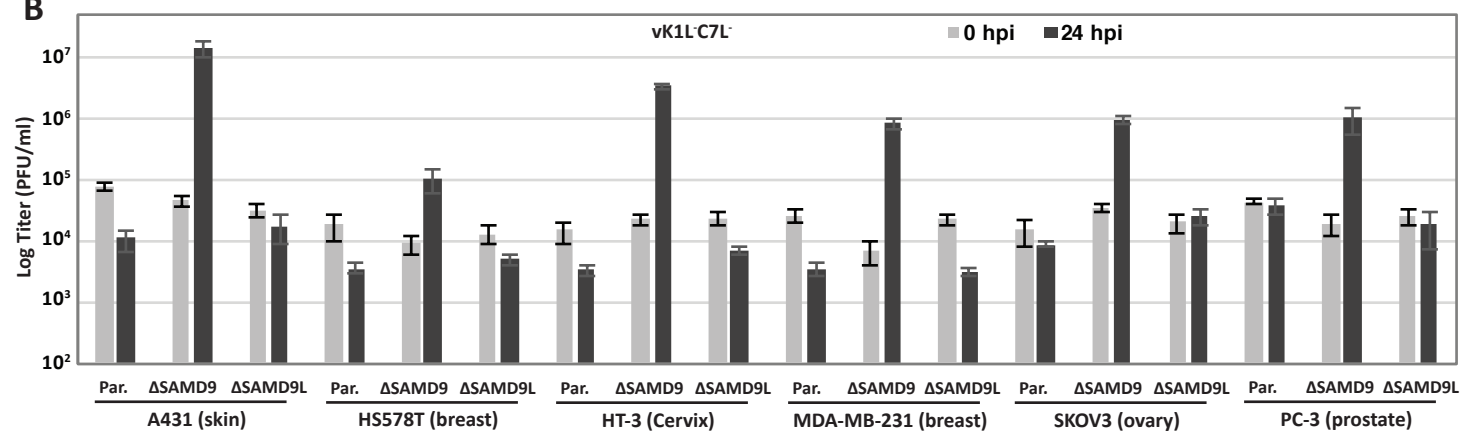**C**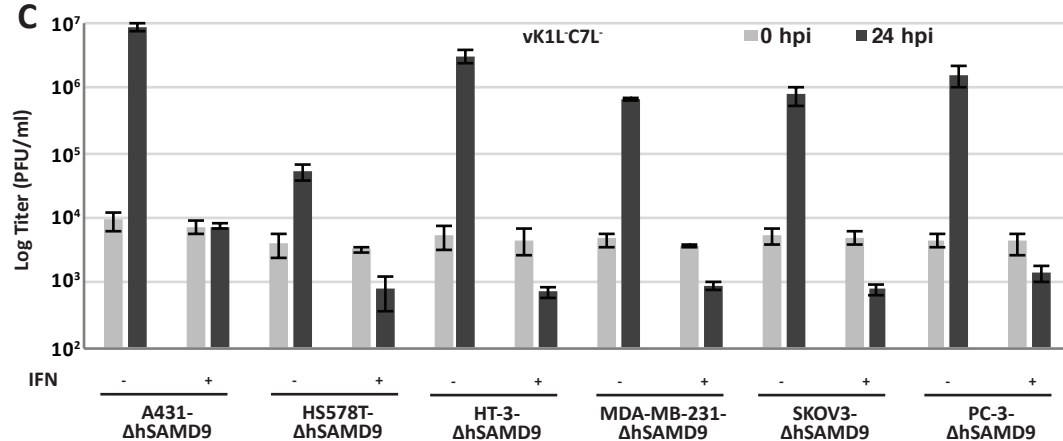

Supplement: S5 Fig — (A). Either hSAMD9 or hSAMD9L was knocked down from various human cells with CRISPR-Cas9 as described in S4 Fig. Pooled knockdown cells were used without clonal selection, and the knockdown was validated by Western blot. Par., parental; Δ9, hSAMD9-knockdown; Δ9L, hSAMD9L-knockdown. (B). Knockdown of hSAMD9 but not hSAMD9L abolished the restriction for vK1L-C7L- in human cells from diverse tissues. The parental and the knockdown cells were infected with vK1L-C7L- at an MOI of 1 PFU/cell. (C). IFN restored host restriction for vK1L-C7L- in hSAMD9-knockdown (ΔhSAMD9) cells. Various ΔhSAMD9 cells were left untreated or treated with IFN-β and infected with vK1L-C7L-. Viral growth was determined by measuring viral titers at 0 and 24 hpi. (PDF) [file ppat.1006884.s005.pdf]

**A**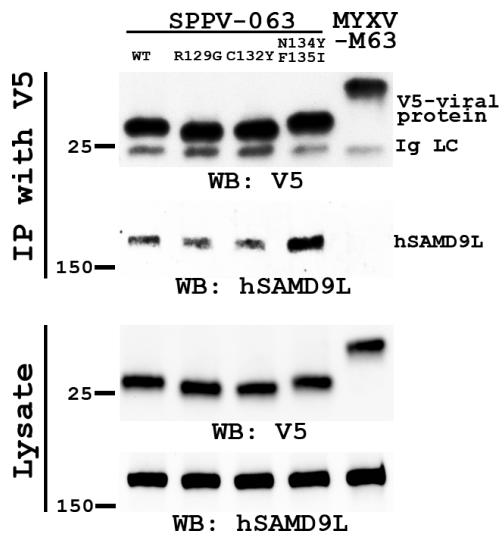**B**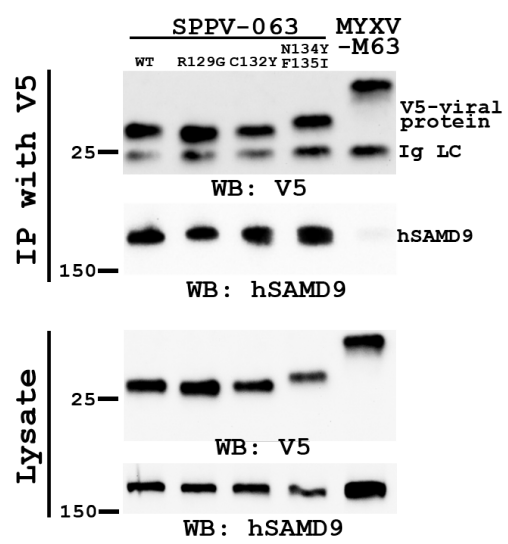

Supplement: S6 Fig — IFN-treated ΔSAMD9 HeLa cells (A) or untreated parental HeLa cells (B) were infected with vK1L-C7L--derived virus that expressed MYXV-M63 (negative control) or SPPV-063 (WT or mutated). The C7 homolog was precipitated with an anti-V5 antibody, and the co-precipitated hSAMD9 or hSAMD9L was detected by Western blot. (PDF) [file ppat.1006884.s006.pdf]
